# Urothelial carcinoma associated 1 promotes trophoblast invasion by regulating MMP9

**DOI:** 10.1186/s13578-019-0341-8

**Published:** 2019-09-23

**Authors:** Hongfang Shao, Feng Jin, Jiangshan Hu, Zhangying Zhu, Fuju Tian, Minfang Tao, Yincheng Teng

**Affiliations:** 1 Center of Reproductive Medicine, Shanghai Jiao Tong University Affiliated Sixth People’s Hospital, 600 Yishan Road, Shanghai, 200233 People’s Republic of China; 2 Department of Gynecology and Obstetrics, Shanghai Jiao Tong University Affiliated Sixth People’s Hospital, 600 Yishan Road, Shanghai, 200233 People’s Republic of China; 3 The International Peace Maternity & Child Health Hospital, Shanghai Jiao Tong University School of Medicine, Shanghai, 200030 People’s Republic of China

**Keywords:** UCA1, Trophoblast invasion, MMP9, Recurrent miscarriage, Early pregnancy

## Abstract

**Background:**

The long non-coding RNA UCA1 is reportedly increased in several human tumors and critical for the cell migration, invasion, or proliferation of several cancer cells. However, the potential roles of UCA1 in trophoblasts at early pregnancy still poorly understood. Here, we sought to unravel the roles of UCA1 in the occurrence of the recurrent miscarriage (RM) disorders.

**Results:**

The knockdown of UCA1 in human HTR-8 trophoblast cell line reduced their cell proliferative and invasive ability. Conversely, the UCA1 overexpression promoted the cell proliferation and invasion of HTR-8 cells. Quantitative RT-PCR screening revealed that UCA1 overexpression significantly enhanced *MMP9*, but not *MMP2*, mRNA expression in trophoblast cells. The overexpression of UCA1 also promoted trophoblast invasion by upregulating MMP9 expression and activity both in vitro and ex vivo. Consistently, *UCA1* and *MMP9* mRNA expression level was notably reduced in placental villi derived from patients with RM diseases.

**Conclusion:**

This study revealed that UCA1 is critical for the regulation of invasive ability in trophoblasts. The abnormal UCA1/MMP9 pathway might result in the impaired trophoblast activities and lead to the development of RM. Our data may also provide a novel angle for the treatment in RM patients.

## Background

The human placenta has been reported to be a unique organ of pregnancy that is critical for the physiological functions of a normal pregnancy. The embryo implantation heavily relies on embryonic hatching, trophoblast formation, appropriate maternal–fetal interactions as well as immune tolerance [1, 2]. During embryo implantation, cytotrophoblasts undergo an epithelial–mesenchymal transition and differentiate into extravillous cytotrophoblasts (EVTs), which are able to migrate into and remodel the maternal tissues (interstitial EVT) and spiral arteries (endovascular EVT) in a uterine to rebuild the uterine vascular system [3]. Abnormal EVT function usually leads to the failure to build up the tight maternal–placental–fetal communication, which is correlated with fetal growth restriction, preeclampsia, and recurrent miscarriage (RM) disorders [4, 5]. In the past, some new therapies to prevent RM diseases have achieved certain clinical success. However, more than 25% of RM patients are still not able to get a successfully conceive resulting from the lack of the efficient treatments [6–8]. Unraveling the underlying mechanisms related to the RM diseases and developing novel strategies for its treatment are critical for improving the successful pregnancy rate in RM patients. The identification of novel and important regulators involving the biological behaviors of trophoblasts will definitely benefit the elucidation of the initiation and progression of RM disorders.

The transcriptional landscape of organisms is much more complicated than originally imagined, as the vast majority of genomic sequences are universally transcribed into both multiple non-coding RNAs (ncRNAs) and protein-coding RNAs [9]. Plenty of studies have demonstrated that protein-coding genes may only account for approximately 1.5–2% of the human genome, while most transcripts are composed of non-coding RNAs, such as long non-coding RNAs (lncRNAs) and microRNAs [10, 11]. Many studies have shown that LncRNAs play an important role in the regulation of cell fate determinations, including cell growth, stem cell pluripotency and embryo development, which further controls the cell functions and related pathological status or disorders in clinic [12–14]. Human urothelial carcinoma associated 1 (UCA1), which is a newly found non-coding RNAs, is highly expressed in tumor cells [15, 16]. Currently, many studies indicate that UCA1 controls the levels of several regulators in some key pathways involved in tumorigenesis. For example, UCA1 overexpression promotes cancer development by activating the mTOR-STAT3 and PI3K-AKT signal pathways, suggesting that lncRNA UCA1 may play a critical role in tumorigenesis, invasion, and metastasis [17]. Moreover, one study also shows that UCA1 fine-tunes the cell cycle in bladder cancer via CREB by a PI3K-AKT-dependent pathway and its expression is negatively correlated with the tumor suppressor of p27 [18, 19]. Meanwhile, studies have revealed that UCA1 is highly transcribed in the embryo, placenta and most fetal tissues during the early development, although its expression is rapidly turned off in most adult tissues right after birth [20, 21]. However, very few studies have been involved in the exploration on the functions of UCA1 in trophoblasts in RM diseases. In this report, we sought to unravel the potential role of UCA1 in RM diseases.

## Methods

### Collection of human embryonic tissues

Fresh villi tissues of placenta from 45 women with RM diseases were collected. The patients’ age was ranged from 23 to 36 years (29.1 ± 6.9 years) and 8–10 weeks of gestation. These placenta villi tissues were obtained in the Department of Obstetrics and Gynecology, Shanghai Jiao Tong University Affiliated Sixth People’s Hospital, from February 2017 to June 2018. The patients accompanying with autoimmune, chromosomal, parental, hormonal, infectious and anatomic disorders, or complications of thyroid abnormalities, diabetes and hypertension, or infection with ureaplasma and chlamydia in cervical mucus, were excluded from this study [19]. Fetal chromosomal abnormalities in RM placenta villi were also excluded.

Additionally, 39 women aged 22–37 years (28.5 ± 7.6 years) with normal gestation were enrolled in health controls (HCs) group. These women were arranged to the HCs group undergoing abortion at the gestation of 6–10 weeks. All HCs group had previous pregnancies without preeclampsia or spontaneous abortion. Written informed consent is requested from the patients involved in current study, which has been approved by the Ethics Committee of Shanghai Jiao Tong University Affiliated Sixth People’s Hospital, Shanghai. The samples from RM patients or HCs were frozen immediately in liquid nitrogen for RNA extraction or fixed with 4% paraformaldehyde for immunostaining. In some cases, the villous explants were freshly cultured. And the primary trophoblasts were isolated before storage.

### Isolation of primary cytotrophoblast cells

Primary trophoblasts were digested by 0.125% trypsin supplemented with DNase I (0.1 mg/mL, Sigma, St. Louis, MO), followed by discontinuous percoll gradient centrifugation, as reported in the previous study [22]. Briefly, the placenta villous samples were rinsed in iced-PBS. The placenta villous samples were digested in a medium of Dulbecco’s Modified Eagle Medium/Nutrient Mixture F-12 (DMEM/F12, Invitrogen, CA, USA) containing DNase I plus trypsin at 37 °C for 20 min for three times. Then, the cytotrophoblasts were isolated by centrifugation at 5% to 65% gradient at steps of 5% and centrifuged at 1000*g* for 10 min. The layer between 35 and 45% aliquots containing the cytotrophoblasts was obtained. The collected primary trophoblasts had a purity of ~ 95%, as examined by FACS for vimentin-negative cells and cytokeratin 7-positive. Purified primary cytotrophoblasts were immediately plated in each well of a 6-well plate at 8.0 × 10^5^ cells/mL, and were further cultured in DMEM/F12 medium containing 20% fetal bovine serum (FBS) and penicillin/streptomycin/gentamicin antibiotics (Invitrogen, CA, USA).

### Cell culture

A human trophoblast cell line derived the first-trimester extravillous trophoblast (EVT), HTR-8/SVneo, was a generous gift provided by Professor. PK Lala (Ontario, Canada) [21]. HTR-8/SVneo cell line was routinely maintained in DMEM/F12 medium containing 10% of FBS and penicillin/streptomycin antibiotics (Invitrogen, CA, USA).

### UCA1 expressing plasmid

To construct an overexpressing UCA1 plasmid, the RNA sequence of human lncRNAs UCA1 was cloned into the modified pcDNA3.1 vector with the primers as following: (1) Forward primer sequence, 5′-ATTGAATTTGACATTCTTCTGGACAATGAGT-3′; (2) Reverse primer sequence, 5′-ACGCGGATCCCTGACTCTTTAGGAAGATTTCT-3′. The pcDNA3.1-UCA1 expressing vector and its empty vector were purified with a commercial Plasmid kit (Qiagen, Hilden, Germany). Then 1.5 μg pcDNA3.1-UCA1 plasmids were transfected into HTR-8 cells when these cells reached 60–70% confluence in a 6-well plate using the jetPRIME^®^ kit following the manufacturer’s instructions (PolyPlus Transfection, Illkirch, France). The pcDNA3.1 empty vector was served as a negative control. HTR-8 cells were treated for the subsequent experiments 24–72 h after transfection.

### Knockdown of UCA1

UCA1 knockdown was performed using three specifics siRNAs that were purchased from GenePharma Inc (Shanghai, China). Briefly, 24 h prior to transfection, HTR-8 cells were cultured in a 6-well plate to reach approximately 40–50% confluence. Then, the trophoblasts were transfected with 100 nmol/L UCA1 siRNA by using Oligofectamine^@^ transfection kit (Invitrogen, CA, USA) in optiMEM medium (Invitrogen, CA, USA) following the manufacturer’s instructions. Random sequence siRNA has been routinely included as a negative control. All the transfected trophoblasts were further used for the subsequent migration and invasion assays 24 h after transfection.

### Cell growth assays

For the cell growth studies, 2 × 10^3^ HTR-8 trophoblast cells were treated with control siRNA, siUCA1#3, empty vector, or UCA1 overexpression vector in each well of a 96-well plate. Cell viability at indicated time points was evaluated with a commercial available Cell Counting Kit-8 (CCK-8; Dojindo Laboratories, Shanghai, China). The absorbance counts were measured at 450 nm by Spectra Max 190 microplate reader (BIO-RAD; Hercules, CA, USA).

### Immunohistochemistry

Immunohistochemical assay was performed according to a previous protocol [23]. Briefly, villous tissue slides were blocked with 5% FBS, followed by the incubation at room temperature at least for 30 min. Then, slides were incubated with anti-MMP9 antibodies (Cat#13667, CST, Massachusetts, USA) at 4 °C for 12 h, followed by the treatment with a HRP/DAB IHC Kit (Abcam, MA, USA). Nuclei of cells were counterstained with Meyer’s hematoxylin (Sigma, MO, USA). The sections were evaluated for the expression of MMP9 using a Leica microscope (Leica, IL, USA).

### Quantification of MMP2 and MMP9

To evaluate the level of MMP2 and MMP9 expression in response to UCA1 knockdown, human HTR-8 cell line was plated and cultured in the 6-well plate, followed by the transfection with control siRNA (siCtrl) or siUCA1 and further cultured for 24 h. The supernatants were collected after centrifugation. The level of MMP2 and MMP9 expression in supernatants were determined by ELISA analysis following the manufacturer’s instructions (R&D Systems, MN, USA).

### Wound healing assay

The wound healing analysis was conducted according to a previous method [24]. In brief, 5 × 10^4^ HTR-8 cells were plated into individual well of a 6-well plate and cultured for 24 h. Then, the trophoblasts were treated with control siRNA, siUCA1 oligos, empty vector, or UCA1 overexpression vector. When the cells reached approximately 85% confluence, the trophoblasts were incubated with Mitomycin C (10 µg/mL, Tocris Bioscience, Bristol, UK) in DMEM/F12 medium for 2 h to eliminate cell proliferative ability. A sterile 200-μL tip was applied to make a scrape across the middle area of each well, followed by washing HTR-8 cells with iced-PBS for three times to remove floating cells or debris. Cells were then cultured in DMEM/F12 medium with 1% FBS for another 16 h. The distance between the edges of the scrape in each well were photographed with a Leica microscope and evaluated in collected photomicrographs. Cell migration was evaluated by comparison of the width of wound closure relative to the initial wound area at 0 h and 16 h.

### Invasion assay

We evaluated trophoblast invasive ability with a Transwell Matrigel invasion method as reported previously [22]. Briefly, 50 µL of Matrigel (dilution 1:4; Corning, New York, USA) was used to coat with the inserts in a 24-well plate (pore size, 8 µm; Corning, New York, USA) on ice. HTR-8 cells or primary trophoblasts freshly isolated from the patients with RM disorders were subjected to the transfection with siCtrl, siUCA1, control vector, and the UCA1 overexpression plasmid and cultured for 48 h. All these transfected cells were plated onto the upper chamber of the transwell insert at 8.0 × 10^4^ cells/200 µL DMEM/F12 medium with 1% FBS. Then, 800 µL of DMEM/F12 medium with 15% FBS were added into the lower chambers. These cells were continually cultured at 37 °C for another 48 h. The inserts were then extensively rinsed with iced-PBS for three–five times, followed by staining with crystal violet (Sigma, MO, USA), and were evaluated under a Leica microscope. The total cell numbers of trophoblasts that had migrated into the low surface of inserts were carefully scored using a microscope. The assay was conducted duplicately and the three repeated experiments were performed independently.

### Statistical analysis

All data are represented as mean ± SD unless otherwise indicated. The software of SPSS 13.0 (IBM, IL, USA) was used for all the statistical analyses. Independent two-tailed Student’s t-test was performed for the comparison of two groups. In some cases, one-way ANOVA with post hoc Tukey’s test was applied for the comparison within multiple groups. Correlations analyses were conducted using Spearman’s rank correlation test. In all the experiments/results, P < 0.05 was set as the statistical significance.

Detailed methods are available in the section of Additional file 1.

## Results

### LncRNA UCA1 enhances trophoblast proliferative and invasive ability in vitro

**Fig. 1:**
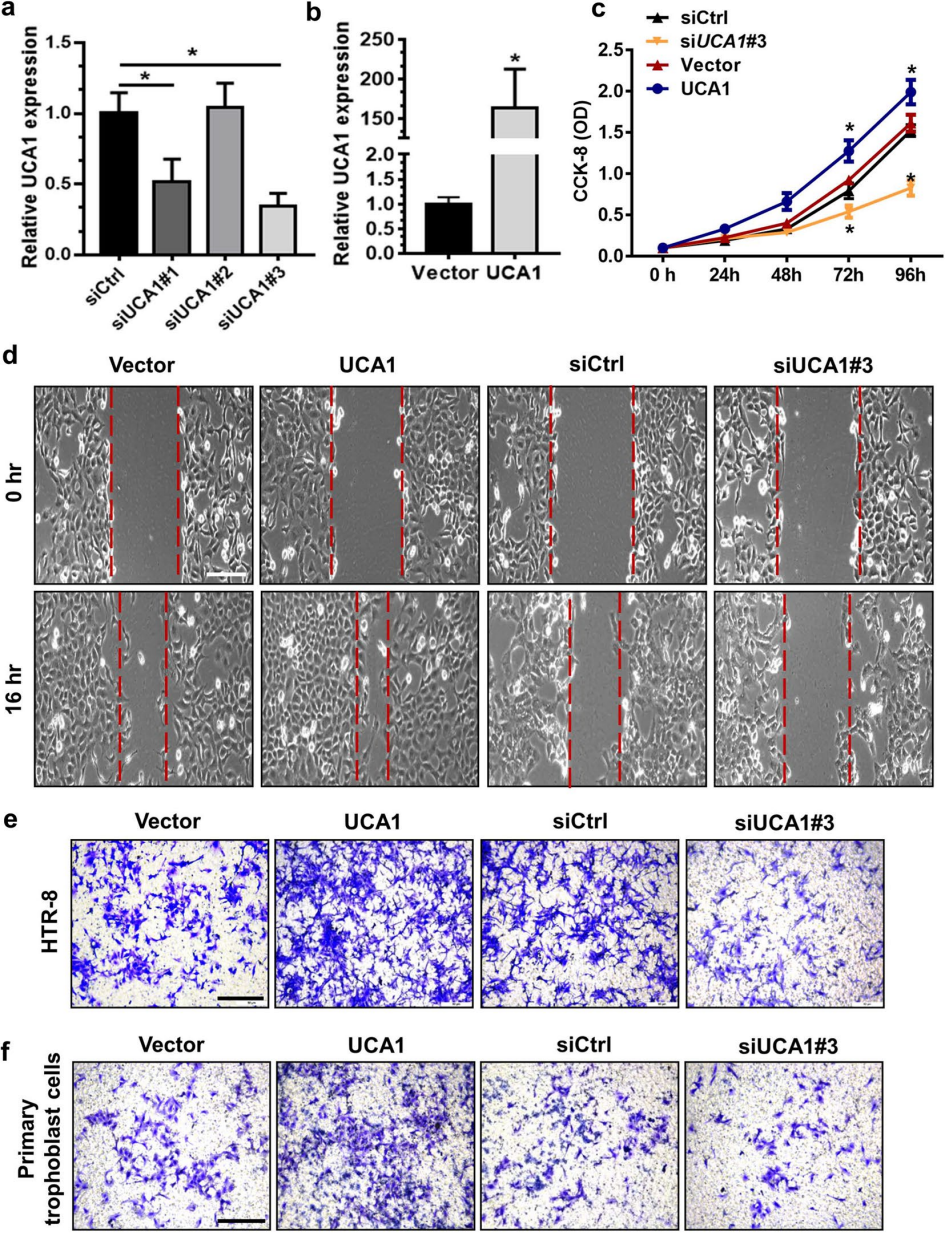
UCA1 regulates trophoblast invasive and proliferative ability in vitro. **a**, **b** Quantitative RT-PCR analysis was utilized for the analysis of UCA1 mRNA expression in HTR-8 cells 48 h after transfection with control siRNA, siUCA1#1, siUCA1#2, siUCA1#3, empty vector, or UCA1-overexpression (OE) plasmid, respectively. *P < 0.05; siRNA vs siCtrl or UCA1-OE vs vector. **c** HTR-8 cells were used for the transfection with control siRNA, siUCA1 #3, empty vector, or UCA1-overexpression vector, respectively. Cell proliferation rate was evaluated by the CCK-8 assay at indicated several time points. *P < 0.05; siRNA vs siCtrl or UCA1-OE vs vector. **d** HTR-8 cells were used for the transfection with siCtrl, siUCA1#3, empty vector or UCA1 overexpression plasmid, respectively. Then the cells were cultured for 24 h to allow them to reach approximately 85% confluence, followed by the analysis for would healing abilities. Overexpression of UCA1 in HTR-8 cells led to the enhanced wound closure ability than that in the ones transfected with empty vector. UCA1-knockdown significantly decreased the extension of wound closure than that in the siCtrl group. Scale bars: 100 μm. **e**, **f** UCA1 overexpression markedly promoted cell invasion of either HTR-8 cells or freshly isolated primary trophoblast cells than that of control ones. Knockdown of UCA1 inhibited cell invasion than that of the siCtrl ones. Scale bars: 100 μm

To explore the functions of UCA1 in trophoblast proliferation and invasion, HTR-8 cell line [23] was used and transfected with the UCA1 siRNAs or UCA1 overexpression vector, followed by culturing for 48 h. As shown in Fig. 1a, b, UCA1 expression was significantly reduced after the siUCA1 treatment but was notably upregulated after treatment with the UCA1 overexpression vector. Furthermore, the Cell Counting Kit-8 (CCK-8) experiment demonstrated that UCA1 overexpression enhanced the HTR-8 cells proliferation, whereas knockdown of UCA1 by siRNA inhibited cells proliferation (Fig. 1c). To further determine whether UCA1 is involved in the regulation of cell migratory or invasive capacities in HTR-8 cells, both Matrigel invasion and wound healing assay were then performed. Our data showed that the UCA1 overexpression obviously promoted cell migration and invasion of both HTR-8 cells and primary trophoblasts. In contrast, the UCA1 knockdown significantly suppressed their migratory and invasive abilities (Fig. 1d–f).

### UCA1 regulates MMP9 expression in trophoblast cells

**Fig. 2:**
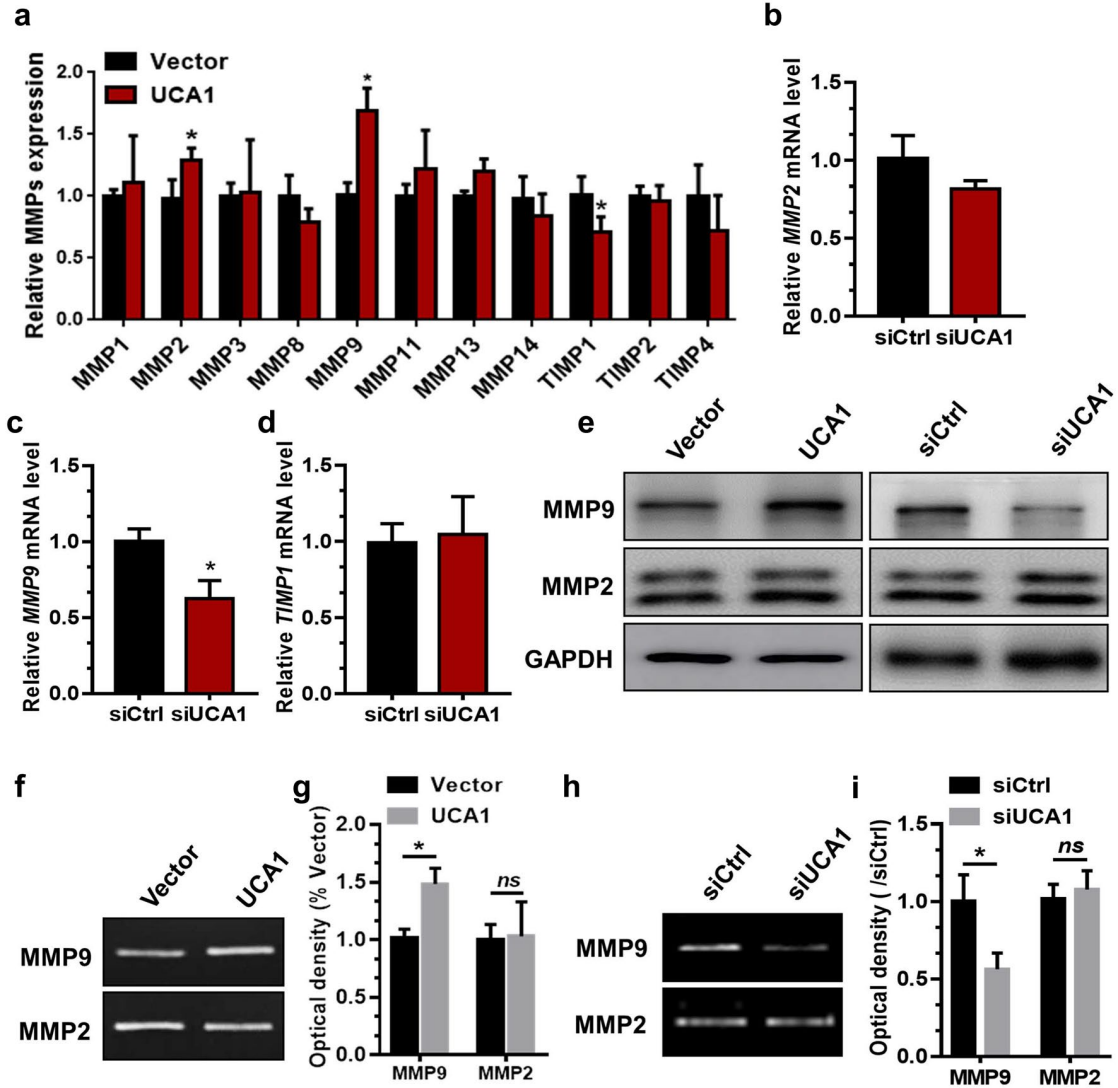
UCA1 promotes the expression of MMP9 in HTR-8 cells. **a** HTR-8 cells were used for the transfection with empty vector or UCA1 overexpression vector. The mRNA levels of the MMPs members were analyzed using quantitative RT-PCR 36 h post-transfection. *P < 0.05; UCA1-OE vs vector. **b**–**d** Levels of *MMP2*, *MMP9*, and *TIMP1* mRNA was determined by quantitative RT-PCR in HTR-8 cells 36 h after transfection with siCtrl or siUCA1. *P < 0.05; siUCA1 vs siCtrl. **e** Levels of MMP9 and MMP2 expression were examined by western blotting analyses in HTR-8 cells 48 h after transfection with siCtrl, siUCA1, control vector, or UCA1 overexpression vector. **f**, **g** Supernatant collected from HTR-8 cells was used for the gelatin zymography assay 48 h after transfection with empty vector or UCA1 overexpression vector. The images of gelatin zymography were captured with a gel imaging system (Tanon 3500R). *P < 0.05; UCA1-OE vs vector. **h**, **i** Supernatant collected from HTR-8 cells was used for the gelatin zymography assay 48 h after transfection with control siRNA or siUCA1. The images of gelatin zymography were captured with a gel imaging system (Tanon 3500R). *P < 0.05; siUCA1 vs siCtrl

We next investigated the underlying mechanisms of UCA1 in the regulation of migration and invasion of trophoblasts. Previous studies have reported that proteolysis of cell extracellular matrix mediated by matrix metalloproteinases (MMPs) is critical for the behaviors of cell invasion. Specifically, MMP2 or MMP9 is well known to be capable of efficiently remodeling the extracellular matrix to facilitate the invasion capacity of trophoblasts [25]. Therefore, quantitative RT-PCR assay was applied to detect the mRNA levels of three *TIMPs* or seven *MMPs* in UCA1-overexpression HTR-8 cells. We observed that the mRNA levels of *MMP2* and *MMP9* mRNA was notably increased in HTR-8 cells, while the *TIMP1* mRNA level was decreased (Fig. 2a). And the mRNA levels of *TIMP2*, *TIMP4*, *MMP1*, *MMP3*, *MMP9*, *MMP10*, *MMP13*, and *MMP14* were not changed after transfection (Fig. 2a). We further downregulated the UCA1 expression by transfecting either siUCA1 or control siRNA into HTR-8 cells. Quantitative RT-PCR data revealed that mRNA level of *MMP9* was much lower in the UCA1-knockdown cells than that of their counterparts. By contrast, level of *MMP2* and *TIMP1* mRNA was not changed in HTR-8 cells from the UCA1 knockdown group (Fig. 2b–d). Using a western blotting assay, we further confirmed that the UCA enhanced MMP9 expression in HTR-8 cells as indicated by UCA1 overexpression or siUCA1 knockdown experiments, while expression of MMP2 was not changed (Fig. 2e). Gelatin zymography assay was also applied to detect the enzyme activities of both MMP2 and MMP9 in the FBS-deprived medium of the HTR-8 cells transfected with either siUCA1 or UCA1 overexpression vector. Our results demonstrated that overexpression of UCA1 enhanced enzyme activity of MMP9, but not MMP2, compared to the control vector-transfected HTR-8 cells (Fig. 2f, g). In contrast, UCA1 knockdown inhibited MMP9 enzyme activity (Fig. 2h, i).

### UCA1 enhances cell invasion of trophoblasts in vitro

**Fig. 3:**
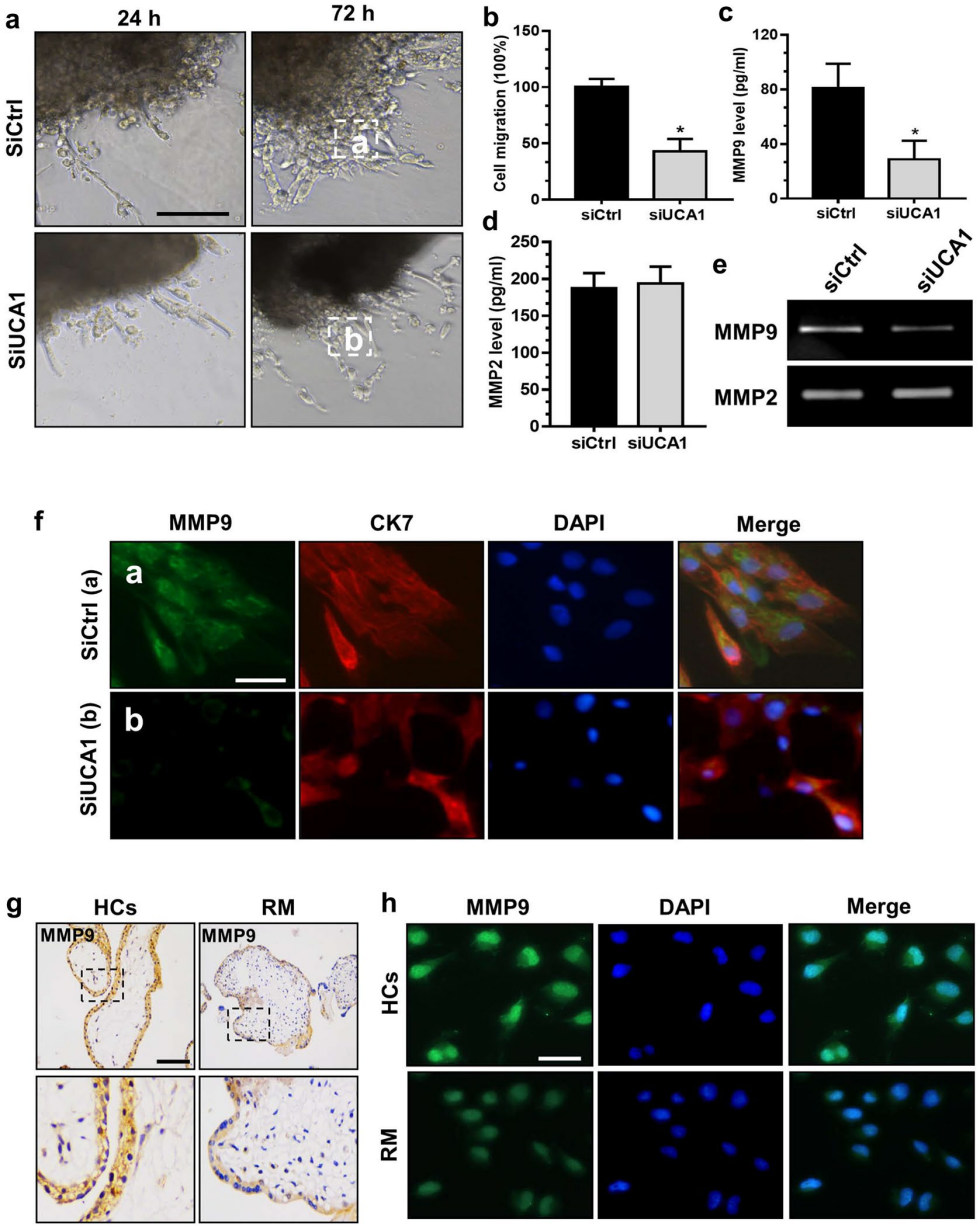
Knockdown UCA1 inhibits trophoblast outgrowth in extravillous explants by downregulating MMP9 expression. **a** Placenta explants from HCs (6–10 weeks) were seeded into Matrigel of a 24-well plate. The villi explants were transfected with siCtrl or siUCA1, and a serial of pictures for explants were individually taken using the microscope 24 h and 72 h after transfection. Scale bars: 250 μm. **b** The outgrowth capacities of the trophoblasts were determined with the ImagePro plus 6.0 software. *P < 0.05; siUCA1 vs siCtrl. **c**–**e** Placenta explants from HCs were seeded into Matrigel of a 24-well plate. The villi explants were then transfected with control siRNA or siUCA1 and cultured for 48 h. The supernatants were collected for ELISA experiments and gelatin zymography experiments. *P < 0.05; siUCA1 vs siCtrl. **f** Whole-mount immunofluorescent staining using anti-MMP9 antibody exhibited a notable decrease of the MMP9 level (green) in siUCA1-knockdown group (b) than that of the siCtrl one (a). Scale bars: 25 μm. CK7 level was shown in red color and nuclei were counterstained with DAPI (blue). **g** Immunohistochemical staining of villous trophoblasts from RM patients (*n* = 15) and HCs (*n *= 15) using anti-MMP9 antibody and a HRP IHC kit. Scale bars: 100 μm. **h** Primary cytotrophoblast cells were freshly collected from the first-trimester villi tissues from either RM patients or HCs, and expression of MMP9 was measured using immunofluorescence staining. Scale bars: 25 μm

To further clarify the function of UCA1 in the cell invasion in trophoblasts in vitro, placenta explants isolated from the first-trimester villi (6–10-week gestation) of the healthy controls (HCs) were seeded into Matrigel pre-coated 24-well plates, followed by the transfection with either siCtrl or siUCA1. The distances of explants tips on the Matrigel were determined at both 24 h and 72 h after transfection. Placenta villi explant tips were found to be anchored in the Matrigel in 24-well plates and started to exhibit outgrowth 24 h after culture. At this time point, no differences were observed between the siUCA1 group and siCtrl one. However, the placenta villi explants transfected with siUCA1 migrated much slower than that of the siCtrl transfected one 72 h later (Fig. 3a, b). ELISA assay also revealed that MMP9 protein level (but not MMP2) was obviously reduced in the siUCA1 group than that of the siCtrl one (Fig. 3c, d). Further, FBS-free medium from the explant treated with siCtrl or siUCA1 was collected, followed by the determination of the enzyme activities of MMP2 and MMP9 proteins as indicated by gelatin zymography assay. Our results showed that siUCA1-transfected explants had much lower activity of MMP-9 (but not MMP2) than that in siCtrl group (Fig. 3e). Immunofluorescent staining of MMP9 also showed that siUCA1 knockdown inhibited MMP9 expression in the explant outgrowth that that of the control siRNA one (Fig. 3f). Moreover, immunohistochemical assay was performed in paraffin-embedded first-trimester villi tissues to evaluate the MMP9 protein level collected from both RM patients and HCs. We observed that MMP9 protein level was markedly reduced in the placenta villi tissues of RM patients than that of the HCs group (Fig. 3g). These findings were further confirmed by the immunofluorescence results, where MMP9 expression level was notably reduced in trophoblasts isolated from patients with RM disorders than those from HCs (Fig. 3h).

### UCA1 is downregulated in villi tissues from RM patients and positively correlated with MMP9 expression

**Fig. 4:**
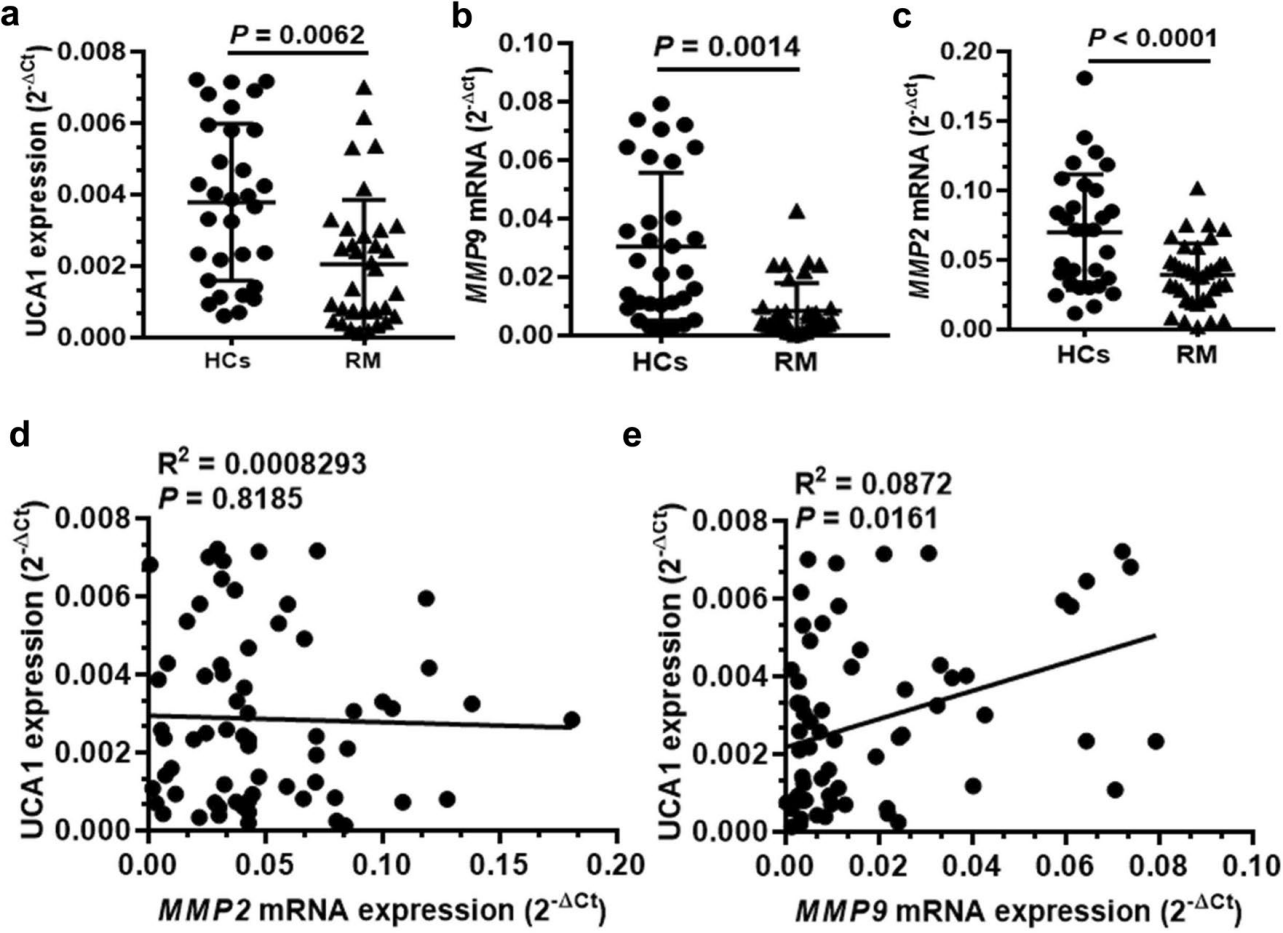
UCA1 expression level is positively correlated with MMP9 level of villi tissues derived from RM patients or HCs. **a**–**c** The mRNA levels of *UCA1*, *MMP2*, and *MMP9* of the villi tissues collected from RM patients (n = 43) or HCs (n = 37) were detected by quantitative RT-PCR. **d** The mRNA level of *MMP2* of the villi tissues isolated from RM patients and HCs was evaluated by quantitative RT-PCR. The potential correlation with the UCA1 level in the villi tissues from RM patients (*n *= 43) and HCs (*n *= 37) were analyzed. **e** The mRNA level of *MMP9* in collected villi tissues of RM patients and HCs was examined by quantitative RT-PCR. The positive correlation with the level of UCA1 expression in RM patients (*n *= 43) and HCs (*n *= 37) were observed

To further determine the potential clinical significance of UCA1 and MMP9 levels, we measured the mRNA levels of *UCA1*, *MMP9* and *MMP2* in villi tissues isolated from RM patients and HCs. Interestingly, our data showed that the expression of UCA1 was obviously attenuated in the placenta villi tissues collected from RM patients than that from HCs. Consistent with the downregulation in *UCA1* level, the level of *MMP9* mRNA expression was markedly downregulated in villous sample from RM patients (Fig. 4a, b). Although the mRNA level of *MMP2* was also decreased (Fig. 4c), linear correlation assay revealed that there was no direct correlation between the UCA1 mRNA level and *MMP2* mRNA expression in villi tissues (Fig. 4d). In contrast, the mRNA level of UCA1 was found to be positively correlated with *MMP9* mRNA expression in primary villi tissues (Fig. 4e). These results support a working model in which UCA1 promotes MMP9 expression in trophoblasts, and the loss of function of UCA1/MMP9 pathways may lead to the occurrence of the RM disorders.

## Discussion

To date, the occurrence frequency of RM diseases significantly increases along with the gestation ages [6]. However, the underlying mechanisms related to RM development remain largely unknown. In current report, our findings provides interesting evidences for the biological functions of UCA1 in trophoblasts during the remodeling of maternal–fetal interface, as well as its tight connections to the occurrence of RM disorders. Lower expression of UCA1 was found in trophoblasts isolated from RM patients. The overexpression of UCA1 enhanced the migratory and invasive abilities in both HTR-8 cells and primary trophoblasts. Mechanistically, we demonstrated that UCA1 regulated trophoblast invasion via promoting MMP9 expression.

A growing body of evidence indicates that lncRNAs are molecules with previously unrecognized biological functions, and lncRNAs play many important roles in several aspects of both embryonic development and tumorigenesis [16, 26]. To date, plenty of experiments have also revealed that there exists a specific link between lncRNAs and placentation. Several lncRNAs, including MALAT1, HOXA11-AS and MEG3, had been demonstrated to be involved in the dysfunction of trophoblasts in RM diseases and pathogenesis of preeclampsia [27, 28]. Moreover, our previous study also demonstrated that HOTAIR promotes the cell migration and invasion of trophoblasts, which further results in the progression of the RM diseases [29]. Although UCA1 has been found to be tightly connected with the development of many cancers [15–17], Wang et al. [20] also found that UCA1 was highly expressed in the villus and placenta. However, the relationship between UCA1 and RM diseases, as well as the downstream targets of UCA1, has not been fully clarified. Here, we demonstrated that UCA1 expression is much lower in the placental villi tissues derived from RM patients than that of HCs. A previous study reported that overexpression of UCA1 enhanced MMP13 expression in the chondrocytes to promote cell proliferation and collagen expression in chondrocytes [30]. Our current data strongly support the role of UCA1 in mediating normal behaviors of trophoblasts. And we revealed that UCA1 can acts as a key regulator of trophoblast invasion at early pregnancy. Recently, Wang et al. [31] showed that UCA1 can increase the metastatic ability of gastric cancer cells via the promotion of Cbl-c-mediated ubiquitination and degradation of GRK2 protein, which in turn enhances the activation of the ERK-MMP9 signaling pathway. Moreover, Yang et al. [32] reported that UCA1 can enhance the expression of downstream targets of the β-catenin-WNT signaling pathways in oral squamous cell carcinoma, such as, MMP9, CCND1, and TCF4. However, whether UCA1 regulates the expression of MMP9 in human trophoblasts through these similar signaling pathways remains unknown. Thus, more detailed studies are warranted to explore the role of UCA1 in the regulation of MMP9 expression in trophoblasts to elucidate the underlying molecule mechanisms.

In vitro and ex vivo *study* has indicated that the processes of the invasion of trophoblasts are correlated with complicated communications involving in the destruction of the extracellular matrix, enhanced cellular adhesiveness and invasive abilities [24, 28]. MMPs are reported to be a family of zinc-dependent endopeptidases, which can efficiently reconstruct the extracellular matrix and fine-tine cell invasion activities [25]. MMP2 and MMP9 had been found to play a specific role in trophoblast invasion, which was indispensable for embryo implantation and placenta development at early pregnancy [33]. Our study demonstrated that UCA1 could increase the production of MMP9, but not MMP2, in both HTR-8 cells and primary trophoblast cells. We also demonstrate that UCA1 is required for the maintenance of the invasion ability of trophoblasts through the upregulation of the MMP9 level, which suggests that MMP9 is a key downstream target of UCA1 in trophoblasts.

## Conclusions

In summary, our study demonstrates that UCA1 plays an important regulator of MMP9 and trophoblast invasion, in addition to its role in the cancer development. UCA1 and MMP9 are downregulated in villi tissues from RM patients, and MMP9 acts as an important mediator of trophoblast invasion. The aberrant UCA1/MMP9 signaling may eventually lead to the acceleration of the RM disorders. Our study provides a novel angle to understand the molecule mechanisms involved in the trophoblast behavior and its connections to the occurrence of RM disorders. Therefore, our data indicate that UCA1 might be a potential diagnosis and therapeutic target for RM diseases.

## Supplementary information

**Additional file 1.**

## Data Availability

All the datasets included and/or analyzed in current report are available from the corresponding author on reasonable request.

## Abbreviations

RM: recurrent miscarriage
lncRNAs: long noncoding RNAs
UCA1: urothelial carcinoma associated 1
EVTs: extravillous cytotrophoblasts
ncRNAs: noncoding RNAs
siRNAs: small interfering RNAs
ECM: extracellular matrix
MMP2/9: matrix metalloproteinase 2/9
