# Supplementary material for: Urothelial carcinoma associated 1 promotes trophoblast invasion by regulating MMP9
Source: Cell Biosci. 2019 Sep 23;9:78. doi: 10.1186/s13578-019-0341-8 (PMC6757381; doi:10.1186/s13578-019-0341-8)
Supplement: Supplementary file 1 — Additional file 1. [file 13578_2019_341_MOESM1_ESM.doc]

**Additional Methods**

**Immunofluorescence Staining**

Primary cytotrophoblast cells from RM patients and healthy controls (HCs) were plated into a 24-well plate. After 24 h, the cells were washed for three times with iced-PBS, and then fixed with 4% PFA for 10 min, followed by staining with anti-MMP9 (dilution 1:200, CST, Massachusetts, USA). The cells were washed with iced-PBS for three times. Then, the cells were incubated with Alexa 488-conjugated secondary antibodies (Invitrogen, CA, USA) for 2 h, followed by the conterstaining with 4',6-diamidino-2-phenylindole. The images were captured by a fluorescent microscope (Leica, IL, USA).

Whole-mount immunofluorescent staining was used to identify the level of MMP9 expression. The villi explants were cultured with Matrigel in a 24-well plate for 3 days, followed by the fixation with 4% PFA for 30 min and washing with iced-PBS for 10 min. The villi explants were further incubated with a blocking buffer for 2 h and then treated with anti-CK7 (dilution 1: 200; Abcam, MA, USA) or MMP9 for 48 h at 4 °C. The villi explants were washed with iced-PBS for three times. Explants were then treated with Alexa 488- or 594-conjugated secondary antibody for 2 h, followed by the conterstaining with 4',6-diamidino-2-phenylindole and evaluated for the expression level.

**Gelatin Zymography**

Gelatinolytic activity of MMP2 and MMP9 was detected with 10% polyacrylamide gels and 0.5 mg/mL gelatin. Briefly, the supernatant of cultured cell was precipitated using TCA Protein Precipitation Kit (Sangon Biotech, Shanghai, China), and total protein level was determined by BCA Kit. The total protein was injected to substrate-gel for electrophoresis. Then the gel was washed with 2.5% Triton X-100 (v/v) twice, treated with 50 mM Tris-HCl (pH 7.5) at room temperature for 30 min to remove the SDS component, and subsequently incubated in a calcium assay buffer at 37 °C for 24 h. Coomassie brilliant blue R250 was used to stain with the gel. The images of gel were taken using imaging system (Tanon, Shanghai, China).

**Villous Explant Culture**

Fifteen-twenty small 3-4 mm villous tissue samples were freshly isolated from villi tissues and seeded in a 24-well plate pre-treated with Matrigel. Then, the explants were cultured with DMEM/F-12 containing 10% FBS (GE Healthcare, Logan, UT) plus penicillin/streptomycin/gentamicin antibiotics at condition of 5% CO_2_/3% O_2_/92% N_2_. The time point when the placenta villi explants were anchored into the Matrigel and began to show the outgrowth was marked as 24 h. The distance of the villous tips was carefully recorded and evaluated until day 3 using Leica SP8 software (Leica, Buffalo Grove, IL). To determine the role of UCA1 in the outgrowth of EVT, UCA1 siRNA (250 nM) or control siRNA (250 nM) was transfected into two wells of explants that were derived from the same placenta, and the transfection efficiency were evaluated 24 h and 72 h after transfection using a fluorescence microscope (Leica, Buffalo Grove, IL).

**Protein isolation and western blotting analysis**

HTR-8 cells were washed twice with iced-PBS and subjected for the protein extraction using RIPA Lysis Buffer (Sigma, St. Louis, MO) on ice. The lysate was centrifuged at 15000 g/min at 4 °C for 25 min. The total protein was detected by a Pierce™ BCA Kit (Rockford, IL, USA). Antibodies against MMP9 (dilution 1:1000; #13667, CST, Massachusetts, USA) and MMP2 (dilution 1:1000, NB200-193, Novus Biologicals, Littleton, USA) were used for the western blotting analysis following the standard protocol. A rabbit antibody against GAPDH (1:5000, EPR16891, Abcam, MA, USA) was used as an internal control.

**Real-time RT-PCR**

TRIzol was applied to extract total RNA of HTR-8 cells or human trophoblasts ([Thermo](http://www.baidu.com/baidu.php?url=iktK00at2yPmH1xXfJoCdvuHZn0v5EZGeo9jnqiTYa6jabOKCBRZ5m6gCcBg6lAwutb9lU8r7Y6LAaK-DmDXxWjyHBDHMgS6L_1qxHUO9ChUbU85zSHxyU20bu1ywMQT8wLNA_3.Db_imLqap5bMlD1FWCRDsdXAjWYe_bD5_eGSW-WknUPMN5YTVZCmtXh1xvIPMW_vIROwzs1IjAHugEvUr1I3eSJxC4TI7Wo6hC_NR2A5jkq_DZKsTZ-wxoHfHjNDlBeRqnpccmCrAJaGv-55jkS_xQvtDrQjkSgVOg9zNtXMIbs42qh1jbozU2qS1uvUEZx9zN3T-xWYvUS1G_tIhkmhPOW_oLIMW3to_MQRze-kl-9h9mo3Srzkf0.U1Yk0ZDqHA-huiRznZw-mv60Ijd_pyu-FHcsIANWp0KGUHYznWR0u1ddugK1n0KdpHdBmy-bIykV0ZKGujYz0APGujYYnjR0UgfqnH0kPdt1njDz0AVG5H00TMfqPHnY0ANGujYknjDvn-tkPjn3g1DsnWn1g1DsnWm3g1b4n1FxnH0sn1PxnH0knWKxnH0znj-xnH0snHIxnH0kP1-xrHf4rNtknj0dnfKBpHYkPHR0UynqnWTknWT4PHwxP1DdPH0YrHfY0Z7spyfqn0Kkmv-b5H00ThIYmyTqn0KEIhsqnHcsQywlg1csPYsVuZGxPjn1QH7xPH01QH7xPHDvQH7xPHDLQywlg1RLnBdbX-tdP1nVnNt4njcVuZGxrH0YQywl0A7B5HKxn0K-ThTqn0KsTjYs0A4vTjYsQW0snj0snj0s0AdYTjYs0AwbUL0qn0KzpWYs0Aw-IWdsmsKhIjYs0ZKC5H00ULnqn0KBI1Ys0A4Y5H00TLCq0ZwdT1Y4nHmzrHRsn10krj0kPHR3PjTL0ZF-TgfqnHfkP1R4PjDYP103P6K1pyfqrHf4PhfzPW0snj0sP10snfKBUjYs0APzm1Yvnjc1ns&us=0.0.0.0.0.0.0) Fisher, [Massachusetts](https://cn.bing.com/search?q=Waltham,+Massachusetts+wikipedia&FORM=LFACTRE), USA) following the manufacturer’s protocol. Total RNA was used to generate cDNA with a Reverse Transcription Kit (Takara Bio, Shiga, Japan) with random and oligo-dT primers. Quantitative RT-PCR was evaluated with a SYBR Green kit. Levels of *UCA1/MMP2/MMP9* mRNA were calculated by the 2^–ΔΔCt^ methods and *GAPDH* mRNA was used as internal control gene. In some cases, *UCA1/MMP2/MMP9* mRNA levels from patients’ samples were evaluated by the 2^–ΔCt^ methods and normalized to the internal *GAPDH*. The primer sequences used in this study were listed in the Supplementary Table 1 as following.

**Table S1. Primer sequences for quantitative RT-PCR**

| **Genes** | **Sequences (5’-3’)** |
| --- | --- |
| MMP1-F | AAGGCCGACCATAACGACG |
| MMP1-R | GCCACATGGCTTAACCCAATG |
| MMP2-F | TCGGAATGGGACAGACCTACT |
| MMP2-R | TCAAAGGGGTCACATTGCTCC |
| MMP3-F | CGGTTCCGCCTGTCTCAAG |
| MMP3-R | CGCCAAAAGTGCCTGTCTT |
| MMP8-F | TGCTCTTACTCCATGTGCAGA |
| MMP8-R | TCCAGGTAGTCCTGAACAGTTT |
| MMP9-F | GGGACGCAGACATCGTCATC |
| MMP9-R | TCGTCATCGTCGAAATGGGC |
| MMP11-F | GGTGTACGACGGTGAAAAG |
| MMP11-R | GCGGTGGAAACGCCAGTAGTC |
| MMP13-F | TCCTGATGTGGGTGAATACAATG |
| MMP13-R | GCCATCGTGAAGTCTGGTAAAAT |
| MMP14-F | CGAGGTGCCCTATGCCTAC |
| MMP14-R | CTCGGCAGAGTCAAAGTGG |
| TIMP1-F | AGAGTGTCTGCGGATACTTCC |
| TIMP1-R | CCAACAGTGTAGGTCTTGGTG |
| TIMP2-F | AAGCGGTCAGTGAGAAGGAAG |
| TIMP2-R | GGGGCCGTGTAGATAAACTCTAT |
| TIMP4-F | ATCTGTGCAACTACATCGAGC |
| TIMP4-R | CGAGATGGTACAGGGTACTGTG |
| UCA1-F | TCGCCACCTACATTAAAGCTAATAT |
| UCA1-R | AGTTTCGGGTCTAGGGTGCAT |
| GAPDH-F | CACTGGGCTACACTGAGCAC |
| GAPDH-R | AGTGGTCGTTGAGGGCAAT |
